# Visualization of Probiotic-Mediated Ca^2+^ Signaling in Intestinal Epithelial Cells *In Vivo*

**DOI:** 10.3389/fimmu.2016.00601

**Published:** 2016-12-16

**Authors:** Takahiro Adachi, Shigeru Kakuta, Yoshiko Aihara, Tomonori Kamiya, Yohei Watanabe, Naomi Osakabe, Naoki Hazato, Atsushi Miyawaki, Soichiro Yoshikawa, Takako Usami, Hajime Karasuyama, Hiromi Kimoto-Nira, Kazuhiro Hirayama, Noriko M. Tsuji

**Affiliations:** ^1^Department of Immunology, Medical Research Institute, Tokyo Medical and Dental University, Tokyo, Japan; ^2^Department of Biomedical Science, Graduate School of Agricultural and Life Sciences, The University of Tokyo, Tokyo, Japan; ^3^Department of Agrobioscience, Graduate School of Agricultural Science, Kobe University, Kobe, Japan; ^4^Biomedical Research Institute, National Institute for Advanced Industrial Science and Technology (AIST), Tsukuba, Japan; ^5^Department of Bio-science and Engineering, Shibaura Institute of Technology, Saitama, Japan; ^6^Laboratory for Cell Function and Dynamics, Advanced Technology Development Group, Brain Science Institute, RIKEN, Saitama, Japan; ^7^Department of Immune Regulation, Tokyo Medical and Dental University, Tokyo, Japan; ^8^Laboratory of Recombinant Animals, Medical Research Institute, Tokyo Medical and Dental University, Tokyo, Japan; ^9^NARO Institute of Livestock and Grassland Science, National Agriculture and Food Research Organization, Tsukuba, Ibaraki, Japan; ^10^Laboratory of Veterinary Public Health, Graduate School of Agricultural and Life Sciences, The University of Tokyo, Tokyo, Japan

**Keywords:** probiotic, Ca^2+^ signaling, intestinal epithelial cell, intravital imaging, *Lactococcus*, *Bacillus subtilis*, small intestine, germ-free mouse

## Abstract

Probiotics, such as lactic acid bacteria (LAB) and *Bacillus subtilis* var. *natto*, have been shown to modulate immune responses. It is important to understand how probiotic bacteria impact intestinal epithelial cells (IECs), because IECs are the first line of defense at the mucosal surface barrier and their activities substantially affect the gut microenvironment and immunity. However, to date, their precise mechanism remains unknown due to a lack of analytical systems available for live animal models. Recently, we generated a conditional Ca^2+^ biosensor Yellow Cameleon (YC3.60) transgenic mouse line and established 5D (*x, y, z*, time, and Ca^2+^) intravital imaging systems of lymphoid tissues including those in Peyer’s patches and bone marrow. In the present study, we further advance our intravital imaging system for intestinal tracts to visualize IEC responses against orally administrated food compounds in real time. Using this system, heat-killed *B. subtilis natto*, a probiotic TTCC012 strain, is shown to directly induce Ca^2+^ signaling in IECs in mice housed under specific pathogen-free conditions. In contrast, this activation is not observed in the *Lactococcus lactis* strain C60; however, when we generate germ-free YC3.60 mice and observe the LAB stimulation of IECs in the absence of gut microbiota, C60 is capable of inducing Ca^2+^ signaling. This is the first study to successfully visualize the direct effect of probiotics on IECs in live animals. These data strongly suggest that probiotic strains stimulate IECs under physiological conditions and that their activity is affected by the microenvironment of the small intestine, such as commensal bacteria.

## Introduction

Food compounds are digested and absorbed through the gastrointestinal tract for nutrition, and probiotic bacteria and polysaccharides affect immunological homeostasis in the gut (1–6). Fermentative lactic acid bacteria (LAB) are aerobic and abundant in the environment and are very often contained in the average diet, consequently composing a major part of small intestinal commensal flora (7–9). LAB, therefore, affect the maturation of host immune cells and intestinal immune homeostasis under normal steady-state conditions (10–12). Oral administration of some LAB strains has been shown to stimulate innate immunity at mucosal sites and to potentiate systemic immune responses against pathogenic bacteria or viruses (13–17). In addition to resident LAB, orally administrated LAB, although inactive, have a substantial effect on the regulation of immunity. We recently described an anti-inflammatory mechanism exclusive to LAB strains. Most LAB strains contain large amounts of double-stranded RNA and are sensed by the endosomal toll-like receptor (TLR) 3 on intestinal dendritic cells to produce interferon-β. This innate sensing procedure contributes to anti-inflammatory and protective immune responses both locally and systemically; therefore, both live and inactive LAB can be utilized as effective probiotics (12). Functional maturation of the immune system is largely dependent on mucosal biological events, and our findings suggest a co-evolutional process through a long-term mutualism between LAB and the immune system. We have demonstrated that *Lactococci* tolerates bile acids and low pH and adheres to human enterocyte-like Caco-2 cells (18). We have not, however, determined the mechanism of interaction between LAB and intestinal epithelial cells (IECs).

Intestinal epithelial cells communicate with commensal microbes and probiotics and potentiate immune responses *via* cytokines and antigen delivery (19). Probiotics trigger signaling pathways in IECs, such as NF-κB and MAP kinase, which affect the immune response and integrity of the mucosal surface barrier. However, it is difficult to monitor their biological events in real time *in vivo*. This issue became an obstacle in our initial study on the interaction between probiotics and IECs. Thus, it would be of great value to develop a reliable analytical system for intravital imaging of IECs.

Calcium ions (Ca^2+^) are universal second messengers performing multiple functions in most cells. In the immune system, stimulation of immunological receptors, including B-cell antigen and cytokine receptors, induces intracellular Ca^2+^ mobilization concomitant with other signaling events such as phosphorylation of cellular substrates (20–24). To visualize Ca^2+^ signaling *in vivo*, we generated a conditional Föster/fluorescent resonance energy transfer (FRET)-based calcium biosensor Yellow Cameleon 3.60 (YC3.60) transgenic mice (25). YC3.60 is a double-chromophore indicator that employs FRET between a cyan fluorescent protein (CFP) and a circularly permuted variant of the yellow fluorescent protein (YFP) Venus (26). Ca^2+^ signaling can be monitored by measuring the ratio of YFP to CFP (YFP/CFP). FRET-based ratiometric indicators including YC3.60 can be corrected for unequal sensor expression and motion-derived changes in fluorescent intensity. Therefore, ratiometric sensors, such as YC3.60, are suitable for *in vivo* whole-body imaging in mice. Accordingly, we have recently established 5D (*x, y, z*, time, and Ca^2+^ signal) live imaging of immunological tissues including those in bone marrow and Peyer’s patches (25).

Here, we applied our system to detect probiotic-mediated Ca^2+^ signaling in IECs *in vivo* and found differences between the two types of Gram-positive probiotic bacteria, *Lactococcus lactis* and *Bacillus subtilis* var. *natto*. Our results suggest, for the first time, that probiotic strains stimulate small IECs *via* intravital observations; in addition, these results facilitate the understanding of probiotic-mediated immunoregulatory mechanisms.

## Materials and Methods

### Mice

The conditional YC3.60 expression transgenic mouse line has been previously described (25). The floxed YC3.60 reporter (YC3.60^flox^) mouse line was crossed with a CD19-Cre mouse line (27), which resulted in CD19^+^ cell-specific YC3.60 expression in YC3.60^flox^/CD19-Cre mice due to the loss of the loxP-flanked neomycin cassette. The YC3.60^flox^ mouse line was crossed with a CAG-Cre (28) mouse line, which expresses the Cre gene ubiquitously. These mice were maintained in our animal facility under specific pathogen-free (SPF) conditions in accordance with the guidelines of the Tokyo Medical and Dental University for animal care. These procedures have been approved by the Committee of the Tokyo Medical and Dental University for animal care.

Germ-free BALB/cA mice were bred at the Laboratory of Veterinary Public Health, the University of Tokyo, and were used as foster mothers. Germ-free animals were kept in flexible vinyl isolators in a room at 24°C, relative humidity of 60%, and 12 h periods of light and dark, and were fed a CMF-pelleted diet (Oriental Yeast Co., Tokyo, Japan) sterilized by γ-irradiation at a dose of 50 kGy. For the generation of germ-free mice with ubiquitous YC3.60 expression, *in vitro* fertilization and cesarean operation were performed as described below. Female mice with ubiquitous YC3.60 expression were superovulated by an intraperitoneal injection of 7.5 IU eCG followed by 7.5 IU hCG at an interval of 48 h. Eggs were collected from sacrificed female mice and fertilized with the sperm of male mice with ubiquitous YC3.60 expression in HTF medium (ARK Resource, Kumamoto, Japan). After overnight culture in the KSOM medium (ARK Resource), two-cell embryos were transferred into the oviducts of pseudopregnant female ICR mice. The estimated delivery date was controlled by a subcutaneous injection of Progehorrmon (Mochida Pharmaceutical Co., Ltd., Tokyo, Japan). The surrogate mothers were sacrificed at the fetal age of 19.5 days by cervical dislocation, and the uterus was aseptically removed with clamps at the top of each uterine horn and the base of the uterus close to the cervix. The uterus was introduced into an isolator for operation through a germicidal trap with 2% peracetic acid solution kept at 37°C. The uterus was cut with scissors, and pups were removed. Their breathing was stimulated, and they were cleaned with dry gauze. After the pups started breathing normally, they were transferred to the isolator with their foster mothers. The germ status was checked once a month. These procedures were approved by the Committee for Care of Laboratory Animals in the Graduate School of Agricultural and Life Sciences at the University of Tokyo.

### Probiotic Bacteria

*Lactococcus lactis* subsp. *cremoris* C60 (29) was cultured in MRS broth (BD Difco) for 20 h at 30°C (late-log phase) at the National Institute of Advanced Industrial Science and Technology (AIST). The bacteria were harvested, washed two times, and resuspended in sterile saline. The suspensions were then heated for 30 min at 70°C (heat-killed) and were stored at −80°C. Heat-killed *B. subtilis* var. *natto* TTCC12 (late-log phase) were kindly provided from Takano Foods Co. Ltd. and were stored at −80°C.

### Flow Cytometry

Calcium ions mobilization was analyzed using flow cytometry. Ca^2+^ mobilization in YC3.60-expressing cells was analyzed by flow cytometry using CyAn ADP™ (Beckman Coulter) as previously described (25). Antibodies with the following specificity of CD19-Alexa647 and B220-Alexa647 (BioLegend) were used.

### Intravital and *In Vitro* Microscope

Intestinal epithelial cells from anesthetized mice were imaged. Small intestinal tracts were surgically opened lengthwise, placed on a cover glass, and immobilized on a microscope stage. For image acquisition, a Nikon A1 laser-scanning confocal microscope with a 20× objective and NIS-Elements AR software was used as previously described (25). We used a dichronic mirrors (DM457/514) and two bandpass emission filters (482/35 for CFP, 540/30 for YFP). YFP/CFP ratio was obtained by excitation at 458 nm. Images of purified spleen cells in PBS were also obtained as above. Acquired images were analyzed with NIS-Elements software (Nikon).

## Results

### Establishment of *In Vivo* Ca^2+^ Signaling Detection System in Intestinal Gut Epithelial Cells

**Figure 1:**
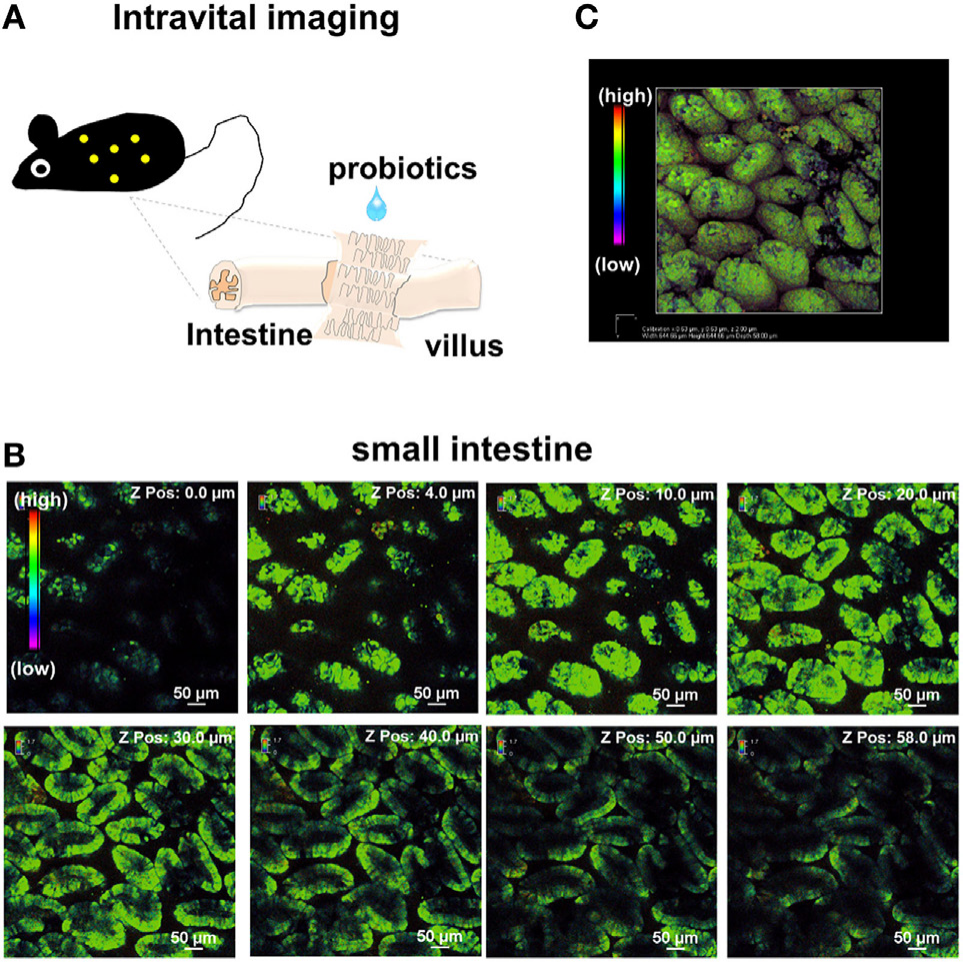
**Structure of intestinal villi in a mouse under ubiquitous YC3.60 expression**. **(A)** Schematic method of intravital imaging of intestinal epithelial cells (IECs). **(B)** *Z*-stack analysis of epithelial cells in the intestinal tract. Intravital imaging of small intestinal villi in the jejunum was performed using confocal lazar microscopy. Ratiometric images (yellow fluorescent protein/cyan fluorescent protein at excitation of 458 nm) are shown. *Z*-stack images of 2-µm intervals up to a depth of 58 µm were obtained. Only representative images are shown. A rainbow parameter indicates relative Ca^2+^ concentration; scale bar, 50 µm. **(C)** 3D structures of small IECs with intracellular Ca^2+^ concentrations. 3D images based on *Z*-stack images **(B)** were obtained using NIS-Elements software. Shown are representative results from three mice.

We previously established an intravital imaging system of Ca^2+^ signaling in lymphoid tissues, such as in Peyer’s patches, spleen, and bone marrow (25). To visualize Ca^2+^ signaling in IECs, we surgically opened the small intestinal tract of the mice with ubiquitous YC3.60 expression, fixed a cover glass on it, and placed it on the stage of the confocal microscope (Figure 1A). Images of the villi in the middle of the small intestine of the mice with ubiquitous YC3.60 expression are shown in Figure 1B. Images of over 50 µm from top of the villi to the basal were obtained. Reconstructed 3D structures showed that almost the entire length of the small intestinal villi could be visualized (Figure 1C). There were no salient differences in intracellular Ca^2+^ concentration among the total epithelial cells, and they included heterogeneous minor subpopulations, such as goblet cells, enteroendocrine cells, and tuft cells (30, 31).

**Figure 2:**
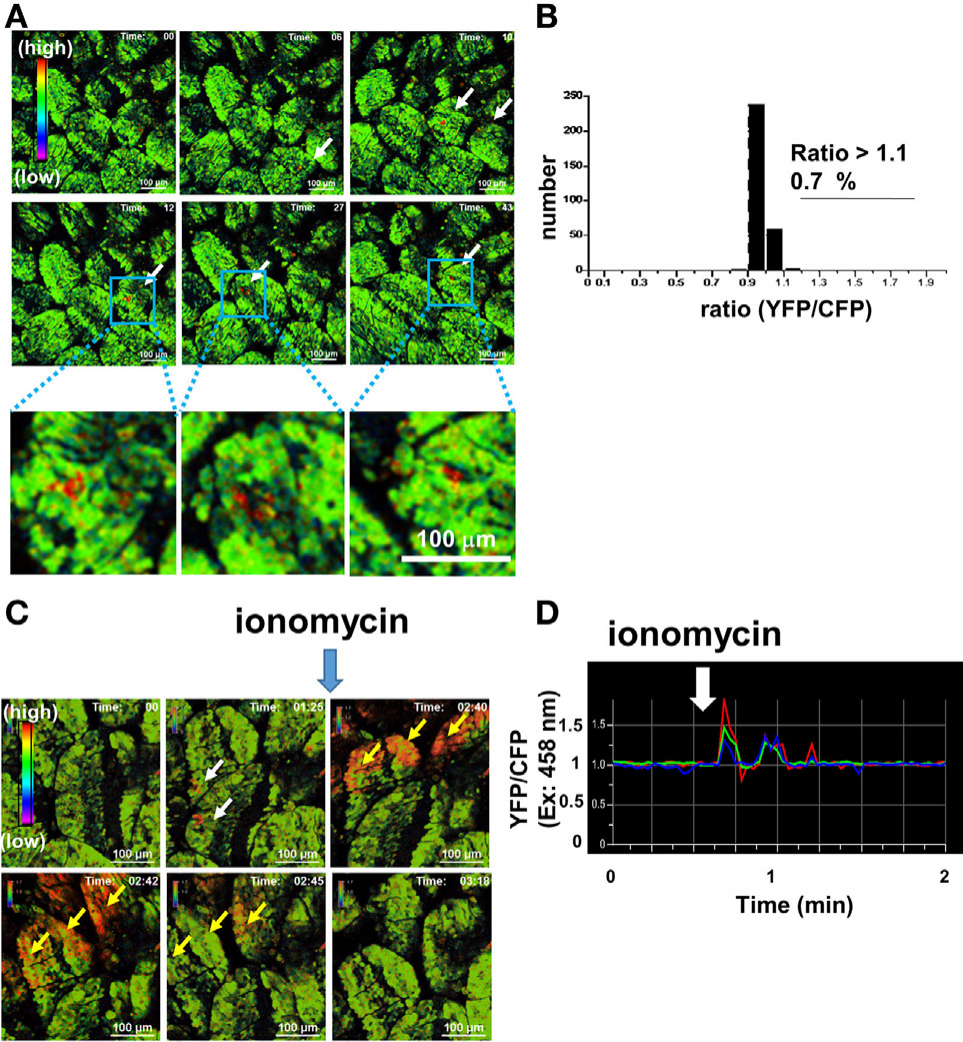
**Intravital Ca^2+^ signaling images mediated by ionomycin in the intestinal tract of a mouse under ubiquitous YC3.60 expression**. **(A)** Representative Ca^2+^ signaling images in the intestinal tract of a mouse under ubiquitous YC3.60 expression without any stimulation. Ratiometric images [yellow fluorescent protein (YFP)/cyan fluorescent protein (CFP) at excitation of 458 nm] are shown. The rainbow parameter indicates relative Ca^2+^ concentration. **(B)** Distribution of time-integrated intracellular Ca^2+^ concentrations of randomly selected regions. *N* = 10; frame = 30. **(C)** Representative Ca^2+^ signaling images in the intestinal tract of a mouse under ubiquitous YC3.60 expression with ionomycin. Ratiometric images (YFP/CFP at excitation of 458 nm) are shown. Five micromolars of ionomycin in PBS were added at the indicated time point. A rainbow parameter indicates relative Ca^2+^ concentration; scale bar, 100 µm; frame = 85. **(D)** Ratiometric intensities (YFP/CFP at excitation of 458 nm) of indicated regions, represented by yellow arrows in **(C)** (*n* = 3), were measured for 2 min at 2-s intervals. Spontaneous Ca^2+^ signals are indicated by white arrows. Shown are representative results from two mice.

Intravital imaging of the IECs showed sporadic but relatively minute Ca^2+^ signaling in some regions under steady-state conditions (Figure 2A). Less than 1% IECs exhibited spontaneous Ca^2+^ signaling (Figure 2B). To determine if the perceptive Ca^2+^ signaling response is observed in this system, we first tested ionomycin, as a positive control, on the stimuli. Upon the addition of ionomycin, transient Ca^2+^ elevation was observed in many IECs (Figures 2C,D). Thus, a system was established to detect *in vivo* real-time Ca^2+^ signaling of IECs.

### Effect of Probiotics on Ca^2+^ Signaling in the IECs of the Mice with Ubiquitous YC3.60 Expression *In Vivo*

**Figure 3:**
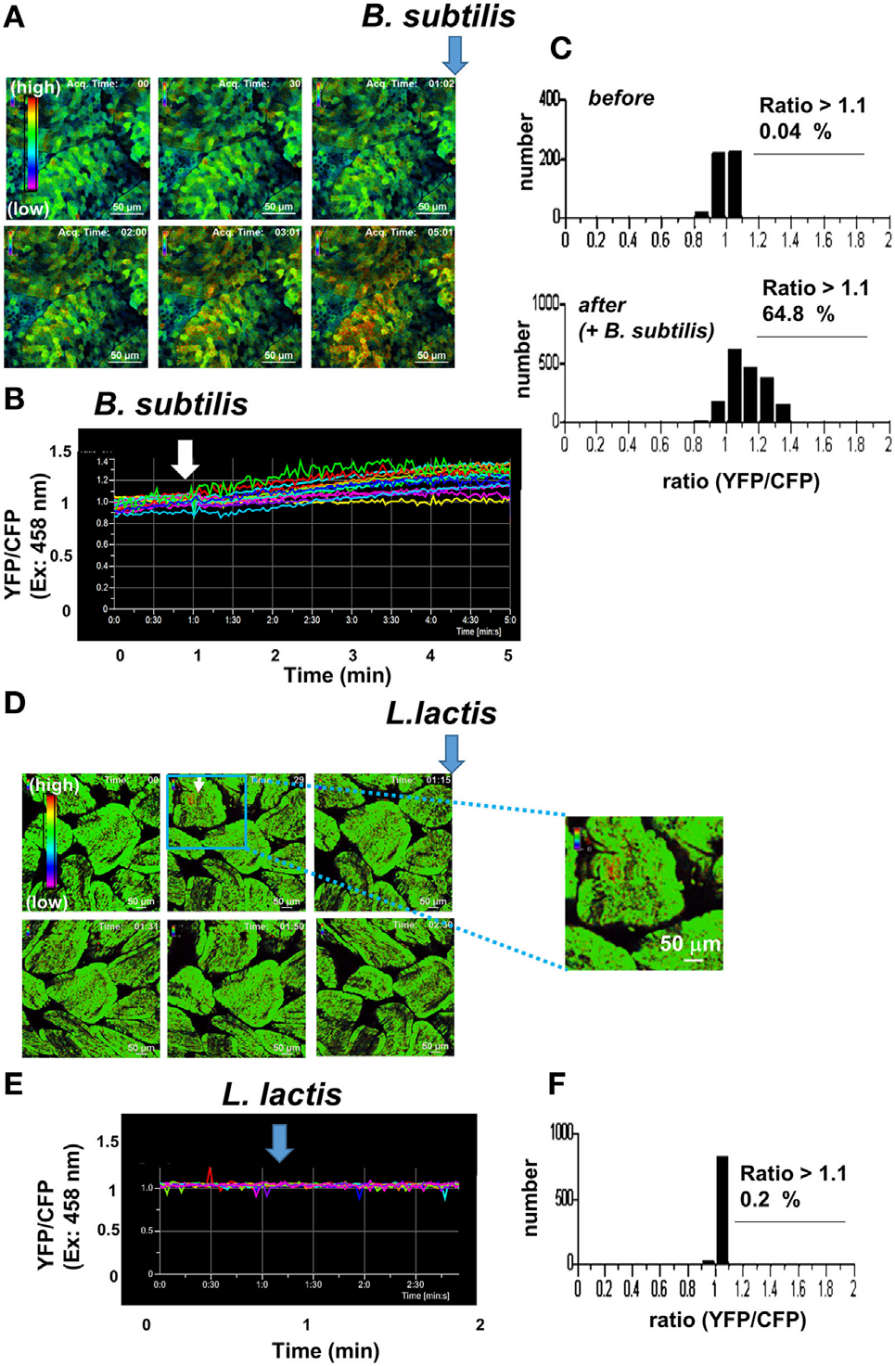
**Intravital Ca^2+^ signaling images mediated by probiotics in the intestinal tract of a mouse under ubiquitous YC3.60 expression**. **(A)** Representative Ca^2+^ signaling images in the intestinal tract of a mouse under ubiquitous YC3.60 expression and specific pathogen-free (SPF) conditions. Ratiometric images [yellow fluorescent protein (YFP)/cyan fluorescent protein (CFP) at excitation of 458 nm] are shown. The 0.1 ml of *Bacillus subtilis natto* in PBS (10^9^ cells/ml) was added at the indicated time point. A rainbow parameter indicates relative Ca^2+^ concentration. **(B)** Time course for fluorescence intensities of YFP/CFP on excitation at 458 nm. Randomly selected regions (*n* = 10) were measured. Scale bar, 50 µm; frame = 151. Spontaneous Ca^2+^ signals are indicated by arrows. **(C)** Distribution of time-integrated intracellular Ca^2+^ concentrations of randomly selected regions before (upper panel) and after (lower panel) stimulation; *n* = 10. **(D)** Representative Ca^2+^ signaling images in the intestinal tract of a mouse under ubiquitous YC3.60 expression and SPF conditions. Ratiometric images (YFP/CFP at excitation of 458 nm) are shown. The 0.1 ml of *Lactococcus lactis* in PBS (10^9^ cells/ml) was added at the indicated time point. **(E)** Time course for fluorescence intensities of YFP/CFP on excitation at 458 nm; frame = 85. **(F)** Distribution of time-integrated intracellular Ca^2+^ concentrations of randomly selected regions; *n* = 10. Shown are representative results from three mice.

*Lactococcus lactis* (18, 29) regulate immune responses by inducing cytokines in dendritic cells and *B. subtilis natto* regulate gut flora and immunity (32–34). We tested whether these probiotics induce Ca^2+^ signaling in IECs. Intravital imaging of IECs exhibited Ca^2+^ signaling upon *B. subtilis natto* treatment (Figure 3A; Video S1 in Supplementary Material). *Bacillus subtilis natto* induced gradual and sustained elevation of intracellular Ca^2+^ concentration in most cells (Figures 3A,B). Figure 3C shows that intracellular Ca^2+^ concentration in IECs was strikingly increased after adding *B. subtilis natto*. Thus, the kinetics of *B. subtilis natto*-mediated Ca^2+^ signaling in IECs is clearly distinct from that observed under steady-state conditions (Figure 2A). One LAB strain, *L. lactis* C60, did not induce Ca^2+^ signaling in IECs except for spontaneous signals (Figures 3D–F). This result is surprising as both *B. subtilis* and LAB are Gram-positive bacteria and well-known probiotics; yet the responses of IECs in SPF mice were distinct in inducing Ca^2+^ signaling. LAB compose a major part of small intestinal commensal flora, and therefore, chronic exposure to the bacteria species may have induced hyporesponsiveness of IECs against LAB.

### LAB Induces Ca^2+^ Signaling in IECs under Germ-Free Conditions

As *Lactococcus* is a related genus of *Enterococcus*, a constituent of gut-resident LAB in the small intestine (7), it may constantly stimulate IECs under steady-state conditions. IECs may refrain from responding to heat-killed *L. lactis* C60 and induce Ca^2+^ signaling due to chronic microbial stimuli by *Enterococci*. We attempted to clarify whether small intestinal microenvironments, especially gut commensal flora, modulate the responsiveness of IECs against LAB.

**Figure 4:**
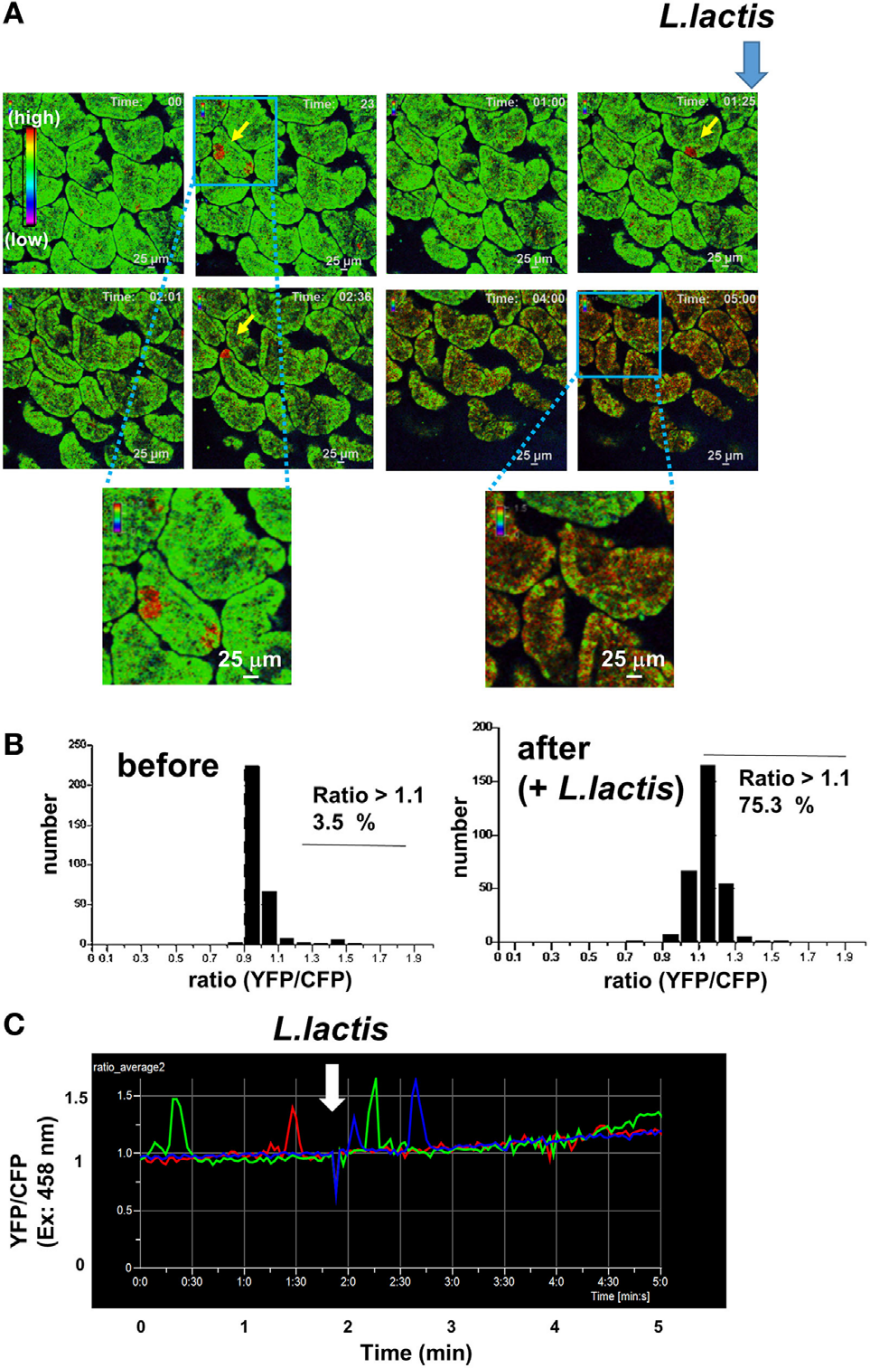
**Intravital Ca^2+^ signaling images mediated by *Lactococcus lactis* in the intestinal tract of a mouse under ubiquitous YC3.60 expression**. **(A)** Representative Ca^2+^ signaling images in the intestinal tract of a mouse under ubiquitous YC3.60 expression and germ-free conditions. Ratiometric images [yellow fluorescent protein (YFP)/cyan fluorescent protein (CFP) at excitation of 458 nm] are shown. The 0.1 ml of *L. lactis* in PBS (10^9^ cells/ml) was added at the indicated time point. A rainbow parameter indicates relative Ca^2+^ concentration. Scale bar, 25 µm; frame = 145. Spontaneous Ca^2+^ signals are indicated by arrows. **(B)** Distribution of time-integrated intracellular Ca^2+^ concentrations of randomly selected regions before (left) and after (right) stimulation. **(C)** Time course for fluorescence intensities of YFP/CFP on excitation at 458 nm in the indicated region is represented by the yellow arrow in **(A)**; *n* = 3. Shown are representative results from three mice.

To this end, we generated germ-free YC3.60 mice to determine whether *L. lactis* can induce Ca^2+^ signaling in IECs in the absence of gut microbiota. As shown in Figure 4 and Video S2 in Supplementary Material, *L. lactis* C60 induced sustained intracellular Ca^2+^ elevation as *B. subtilis natto*, indicating that *L. lactis* C60 can stimulate IECs directly in the absence of gut microbiota. Many IECs were stimulated by adding *L. lactis* under germ-free conditions (Figure 4B). Furthermore, the IECs in germ-free mice exhibited sporadic Ca^2+^ signals under steady-state conditions regardless of *L. lactis* stimulation (Figures 4A,C). The frequency of sporadic Ca^2+^ signals in germ-free mice (Figure 4B, left panel) is higher than that in the SPF mice (Figure 2A).

### *L. Lactis* Induces Ca^2+^ Signaling in B Cells *In Vitro*

**Figure 5:**
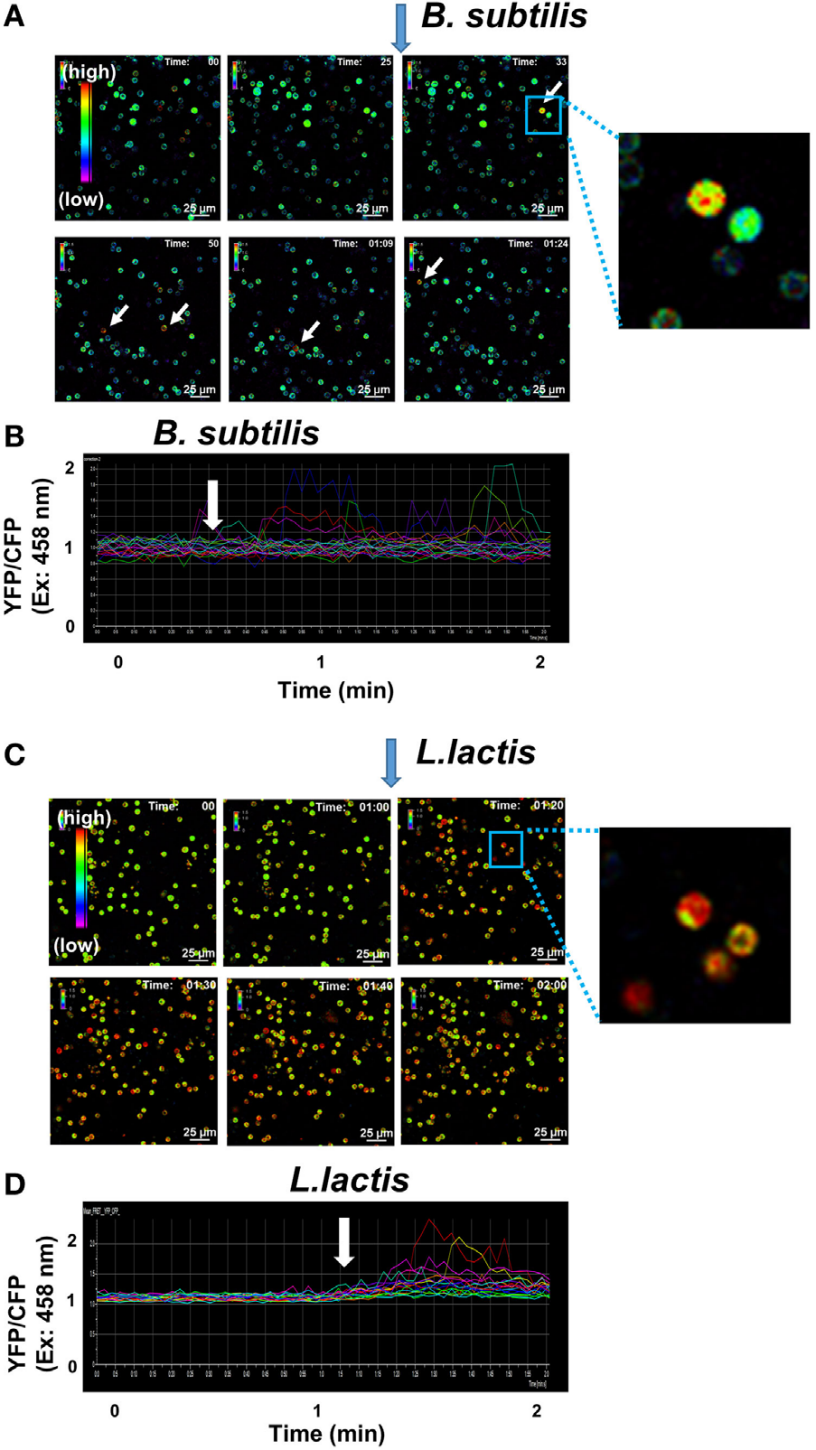
**Ca^2+^ signaling images mediated by *probiotics* in B cells *in vitro***. **(A)** Representative Ca^2+^ signaling images in spleen B cells form CD19-Cre/YC3.60 mice. Ratiometric images [YFP/cyan fluorescent protein (CFP) at excitation of 458 nm] are shown. Ten microliters of *Bacillus subtilis natto* in PBS (10^9^ cells/ml) were added to the cell culture (0.2 ml) at the indicated time point. A rainbow parameter indicates relative Ca^2+^ concentration. **(B)** The time course for fluorescence intensities of YFP/CFP on excitation at 458 nm in the cells (*n* = 15). Scale bar, 25 µm; frame = 56. **(C)** Representative Ca^2+^ signaling images in spleen B cells of CD19-Cre/YC3.60 expression in mice. Ratiometric images (YFP/CFP at excitation of 458 nm) are shown. Ten microliters of *Lactococcus lactis* in PBS (10^9^ cells/ml) were added to the cell culture at the indicated time point. A rainbow parameter indicates relative Ca^2+^ concentration. **(D)** The time course for fluorescence intensities of YFP/CFP on excitation at 458 nm in the cells (*n* = 15). Scale bar, 25 µm; frame = 61. Shown are representative results of three experiments.

Probiotics directly stimulate various immune cells such as dendritic cells, macrophages, NK cells, and T cells (11, 35). We prepared primary B cells from the spleens of YC3.60^flox^/CD19-Cre mice and stimulated with *B. subtilis natto* (Figures 5A,B) or *L. lactis* (Figures 5C,D). Upon stimulation, both *B. subtilis natto* and *L. lactis* induced Ca^2+^ mobilization in primary B cells (Figure 5), confirming their direct stimulation of B cells. Time-lapse observation of single cells was useful to clarify heterogeneity in the kinetics of Ca^2+^ signaling (Figures 5B,D).

## Discussion

Here, we establish a real-time visualization system of Ca^2+^ signaling in small IECs *in vivo* to monitor food signals. By using this system, we show that *B. subtilis natto* triggers Ca^2+^ signaling in IECs and that *L. lactis* evokes Ca^2+^ signaling in gut tissue under germ-free conditions but not under SPF conditions. These results suggest that gut microbiota regulate their responses against orally administered probiotics.

In this study, we successfully visualize probiotic-mediated Ca^2+^ signaling in IECs based on the 5D (*x, y, z*, time, and Ca^2+^) live imaging system in YC3.60 mice (25). A calcium biosensor YC3.60 is a double-chromophore indicator that employs FRET between CFP and YFP mutants (Venus) (26). Ca^2+^ signaling can be monitored by measuring YFP/CFP when CFP is excited. Motion-induced artifacts or unequal biosensor expression are corrected by an internal control in the denominator of the ratio. Thus, the ratiometric sensor YC3.60 is suitable for *in vivo* imaging of the gut, which exhibits vigorous motion with peristalsis in addition to beating and breathing. Moreover, 5D live imaging of tissues enables time-lapse monitoring of dynamic Ca^2+^ signaling in single cells, cell–cell interactions, and other segments of interests. Response of these tissues is quantified and integrated over the desired time span as shown in Figures 2B, 3C,F and 4B.

Probiotic *B. subtilis natto*, but not *L. lactis*, triggers Ca^2+^ signaling in the gut epithelium in SPF mice, although both of them are capable of stimulating spleen B cells from mice with the same microbial conditions. Since *L. lactis* C60 stimulates IECs in the absence of gut microbiota in germ-free mice, the gut microbiota may shape the responsiveness of IECs against LAB.

Intestinal epithelial cells express a series of pattern recognition receptors (PRRs) including TLRs, nucleotide-binding sites, leucine-rich repeat-containing receptors, and retinoic acid-inducible gene-I-like receptors (36). Bacterial components stimulate these PRRs and regulate IRFs, NF-κB, and/or the MAP kinase signaling pathway. One study showed that IECs from germ-free mice show a reduction in TLR expression (37). Another study reported that immunobiotic strains regulate the expression and activity of TLRs in IECs (38). These findings suggest that microbial conditions shape the homeostatic regulation of functional PRRs. Since *Lactococcus* is similar to *Enterococcus*, a major member of small intestinal commensal LAB, probably IECs, at least in part, is hyporesponsive to this symbiotic genus of bacteria. We hypothesize that due to this hyporesponsiveness, further stimulation with *L. lactis* does not induce visible Ca^2+^ signaling despite the expression of PRRs on the IECs. As reported, probiotic strains tolerate IECs (38, 39); such causal relationships may also explain the frequent and sporadic Ca^2+^ signaling observed in IECs of germ-free mice under steady-state conditions (Figure 4). The molecular mechanisms underlying these observations, however, remain unclear.

We find that IECs exhibit sporadic transient Ca^2+^ signaling, although we do not know the precise mechanism of this action. The signals appear to be more striking in the germ-free mice than in the SPF mice, suggesting that these signals are mediated by the gut microbiota or endocrine systems (40).

B cells also express PRRs and are directly stimulated by microbial components (41). Although *L. lactis* C60 fails to induce Ca^2+^ signaling in IECs under SPF conditions, it strongly induces Ca^2+^ signaling in spleen B cells from SPF mice. In contrast, *B. subtilis natto* induces Ca^2+^ signaling in both IECs *in vivo* and B cells *in vitro*. It is not known whether the different reactivity against two types of probiotics can be attributed to the difference in the methods between the intravital and *in vitro* assay, to the skewed influence of stimuli including commensal flora and diet on local and systemic immune cells, or to the outcome of strain difference of probiotics. Additional studies are required to evaluate these results further.

Understanding the function of IECs is important to evaluate immune responses, since stimulated IECs produce cytokines and/or chemokines (11, 23). Goblet cells and M cells deliver antigens to dendritic cells (42, 43). Beneficial probiotic signaling may be transferred to immune cells through these mechanisms, resulting in the regulation of immune tolerance or response. In this study, we show that an intravital imaging system using YC3.60 mice allows for the detection of real-time activation of IECs by probiotics. This system is proven here to be a powerful method for not only clarifying the effects of probiotics on epithelial cell–immune cell communication with stoichiometries but also detecting a subtle disorder before pathological onset (25) and developing preventive and therapeutic strategies with probiotics.

## Ethics Statement

YC3.60 mice were maintained in our animal facility under SPF conditions in accordance with the guidelines of the Tokyo Medical and Dental University for animal care. IECs of anesthetized mice were imaged. Small intestinal tracts were surgically opened, immobilized on a microscope stage, and maintained. Then, images were obtained by a confocal laser microscopy. These procedures have been approved by the Committee of the Tokyo Medical and Dental University for animal care. Germ-free BALB/cA mice used as foster mothers were bred at the Laboratory of Veterinary Public Health, the University of Tokyo. All the germ-free animals were kept in flexible vinyl isolators in a room with 24°C, relative humidity of 60% and 12-h periods of light and dark and fed CMF-pelleted diet (Oriental Yeast Co., Tokyo, Japan) sterilized by γ-irradiation at dose of 50 kGy. For the generation of germ-free ubiquitous YC3.60 expression mice, *in vitro* fertilization and caesarean operation were performed as described below. Female ubiquitous YC3.60 expression mice were superovulated by intraperitoneal injection of 7.5 IU eCG followed by 7.5 IU hCG at an interval of 48 h. Eggs were collected from sacrificed female mice and fertilized with sperm of male ubiquitous YC3.60 expression mice in HTF medium (ARK Resource, Kumamoto, Japan). After over night culture in KSOM medium (ARK Resource), two-cell embryos were transferred into oviduct of pseudopregnant female ICR mice. Estimated delivery date was controlled by subcutaneous injection of Progehorrmon (Mochida Pharmaceutical Co., Ltd., Tokyo, Japan). The surrogate mothers were sacrificed at the fetal age of 21st day by cervical dislocation and “uterine package” was aseptically removed with clamps at the top of each uterine horn and the base of the uterus close to the cervix. The “uterine package” was introduced into isolator for operation through germicidal trap with 2% peracetic acid solution kept at 37°C. Then, “uterine package” was cut open with scissors and pups were taken out. The pups were stimulated breathing while cleaning them with dry gauze. After the pups started breathing normally, pups were transferred to the isolator with foster mothers. The germ-free status was check once a month. These procedures have been approved by the Committee for Care of Laboratory Animals in the Graduate School of Agricultural and Life Sciences at the University of Tokyo. There is no additional consideration.

## Author Contributions

KH, NT, YA, NO, AM, SY, HK, and TA designed the research; TA and NT wrote the manuscript; KH, SK, YA, TU, NH, TK, YW, HK-N, SY, and TA performed the experiments, analyzed the data, and prepared the figures.

## Conflict of Interest Statement

The authors declare that the research was conducted in the absence of any commercial or financial relationships that could be construed as a potential conflict of interest.

## Supplementary Material

Video S1 **Intravital Ca^2+^ signaling images mediated by *Bacillus subtilis natto* in the intestinal tract of a mouse under ubiquitous YC3.60 expression and specific pathogen-free conditions**. *B. subtilis natto* in PBS (10^9^ cells/ml) in PBS was added at the time point of 1 min 30 s. A rainbow parameter indicates relative Ca^2+^ concentration. The time course for fluorescence intensity of yellow fluorescent protein/cyan fluorescent protein on excitation at 458 nm was performed. Scale bar, 50 µm; frame = 151.

Video S2 **Intravital Ca^2+^ signaling images mediated by *Lactococcus lactis* in the intestinal tract of a ubiquitous YC3.60 expression mouse under germ-free conditions**. *L. lactis* in PBS (10^9^ cells/ml) was added at the time point of 1 min 50 s. A rainbow parameter indicates relative Ca^2+^ concentration. The time course for fluorescence intensity of yellow fluorescent protein/cyan fluorescent protein on excitation at 458 nm was performed. Scale bar, 25 µm; frame = 145.
